# Nationwide cross-sectional survey of patients with relapsing polychondritis in 2019 demonstrates reduction of airway involvement compared with that in 2009

**DOI:** 10.1038/s41598-021-04493-0

**Published:** 2022-01-10

**Authors:** Jun Shimizu, Yoshihisa Yamano, Kimito Kawahata, Noboru Suzuki

**Affiliations:** 1 Department of Immunology and Medicine, St. Marianna University School of Medicine, Sugao 2-16-1, Miyamae-ku, Kawasaki, 216-8511 Japan; 2 Institute of Medical Science, St. Marianna University School of Medicine, Kawasaki, 216-8511 Japan; 3 Division of Rheumatology and Allergology, St. Marianna University School of Medicine, Kawasaki, 216-8511 Japan

**Keywords:** Diseases, Rheumatology, Signs and symptoms

## Abstract

We conducted retrospective cohort studies of patients with relapsing polychondritis (RP) twice in 2009 and 2019, using a physician questionnaire. We compared the patients’ clinical statuses between the years. Age and gender were comparable between the two surveys. Mean disease duration was longer in 2019 survey (8.3 years) than that in 2009 survey (4.8 years, P < 0.001). The mortality rate declined in 2019 survey compared with those in 2009 survey (from 9.2 to 1.6%, P < 0.001). Incidence of airway involvement decreased in 2019 survey compared with that in 2009 survey (from 49 to 37%, P = 0.012). In 2019 survey, we found more frequent use of biological agents and immunosuppressants in patients with airway involvement. When we focused on RP patients with airway involvement, physicians in 2019 chose methotrexate and calcineurin inhibitors preferentially, compared with azathioprine and cyclophosphamide. Of note is that increased use of infliximab was observed in RP patients with airway involvement, but not in those without. Reduction of airway involvement and mortality in patients with RP was observed in 2019 survey. The reduction may associate with the frequent use of biologics including infliximab in RP patients with airway involvement.

## Introduction

Relapsing polychondritis (RP) is a multisystemic and inflammatory condition affecting cartilaginous tissues and other connective tissues^1^. Several epidemiological studies demonstrated that respiratory complications were the leading causes of death in patients with RP^2–4^.

We conducted an epidemiological survey of RP patients in 2009 (termed as 2009 survey) using a physician questionnaire and collected the clinical information of 239 patients^5^. From the data, we estimated that the number of RP patients was approximately 400–500 in Japan^5^. We found that 50% patients had airway involvement at the time of the last follow-up and respiratory failure and pulmonary infection were major causes of death^5^. In France airway chondritis were found in 50% patients^4^.

Methotrexate was reported to be useful for the treatment in patients with RP^5,6^. A recent study demonstrated that TNF inhibition improved clinical and laboratory parameters, such as respiratory symptoms and results of pulmonary function test, in RP patients with airway involvement^7^.

In the 1980s, the 10-year survival rate in patients with RP was reported to be approximately 50%^3^. Recent studies revealed that 10-year and 8-year survival rates were 91%^4^ and 94%^6^, respectively.

We here conducted the second nationwide cohort study and obtained clinical information of 190 patients with RP in 2019 (termed as 2019 survey).

We analyzed the information, comparing with that of 2009 survey, focusing on medical treatment on airway involvement in RP patients.

## Results

### Demographic and clinical information elucidated in 2019 survey

We collected newly anonymous information of 190 RP patients in 2019 survey, and the demographic and clinical information were compared with 239 anonymous patient data of 2009 survey (Table 1). The age, age at onset, male–female ratio were comparable between 2019 and 2009 survey.

**Table 1:** Demographic and clinical data of patients with relapsing polychondritis in 2019 survey.

	2009 survey	2019 survey	P-value
Patient numbers	239	190	
**Demographic data**			
Age (year)	57.9 ± 1.1	58.4 ± 1.2	0.72
Age at onset (year)	52.8 ± 1.1	50.3 ± 1.2	0.12
Disease duration (year)	4.75 ± 0.3	8.31 ± 0.6	< 0.001*
Male–female ratio	1.13:1	1.13:1	1.00
**Cumulative incidence of clinical complications, % (number of patients)**			
Ear	78.2 (187)	83.2 (158)	0.31
Nasal	32.6 (78)	26.3 (50)	0.16
Inner ear	26.4 (63)	22.6 (43)	0.36
Joint	38.5 (92)	46.8 (89)	0.082
Eye	48.1 (115)	43.2 (82)	0.31
Airway	49.0 (117)	36.8 (70)	0.012*
Skin	13.8 (33)	5.8 (11)	0.009*
Cardiovascular	7.1 (17)	8.4 (16)	0.61
CNS	11.7 (28)	3.2 (6)	0.001*
Renal	6.7 (16)	7.9 (15)	0.63
**Mortality rates, % (number of patient deaths)**			
	9.2 (22)	1.6 (3)	< 0.001*
**Medication use, % (number of patients)**			
Steroids	91.2 (218)	91.1 (173)	0.95
Immunosuppressants	37.2 (89)	59.5 (113)	< 0.001*
Biologic agents	5.0 (12)	14.2 (27)	0.001*
**Surgical intervention, % (number of patients)**			
Tracheotomy	17.6 (42)	2.6 (5)	< 0.001*
Stenting	9.2 (22)	1.6 (3)	< 0.001*

*Significant differences were observed between 2019 and 2009 survey (P < 0.05).

The disease duration in 2019 survey was significantly longer than that in 2009 survey (P < 0.001).

Incidence of airway involvement (70/190 patients in 2019 survey, 36.8%; 117/239 patients in 2009 survey, 49.0%; P = 0.012), skin involvement (11/190 patients in 2019 survey, 5.8%; 33/239 patients in 2009 survey, 13.8%; P = 0.010), and CNS involvement (6/190 patients in 2019 survey, 3.2%; 28/239 patients in 2009 survey, 7.1%; P = 0.001) decreased in 2019 survey (Table 1). Incidence of nasal, inner ear, eye, ear, joint, cardiovascular, and renal involvement were almost comparable between the two surveys (Table 1).

The mortality rate declined (P < 0.001) in 2019 survey compared with that in 2009 survey (Table 1). 3 patients died (one pulmonary infection, one systemic vasculitis, and one uterine cancer) in 2019 survey where 190 patients were enrolled. 22 patients (9.2%) died in 2009 survey of 239 patients, including respiratory failure (8 patients) and pulmonary infection (4 patients). Numbers of RP patients treated with tracheotomy and/or stenting decreased in 2019 survey (P < 0.001, Table 1).

In 2009 survey, age (P = 0.30), age at onset (P = 0.61), disease duration (P = 0.20), and gender (P = 0.23) did not associate with clinical prognosis^8^. Similarly, age (P = 0.12), age at onset (P = 0.11), disease duration (P = 0.84), and gender (P = 0.74) did not associate with clinical prognosis in 2019 survey.

### Frequent use of immunosuppressants and biologic agents for the treatment of RP patients with airway involvement in 2019 survey

RP patients were considered to be categorized into two groups, patients with airway involvement and those without airway involvement^8^, and thus, we were interested in a reduction of airway involvement of this study. It is possible that therapeutic advances may have an association with the reduction. We therefore focused on medication of 2019 survey.

**Figure 1:**
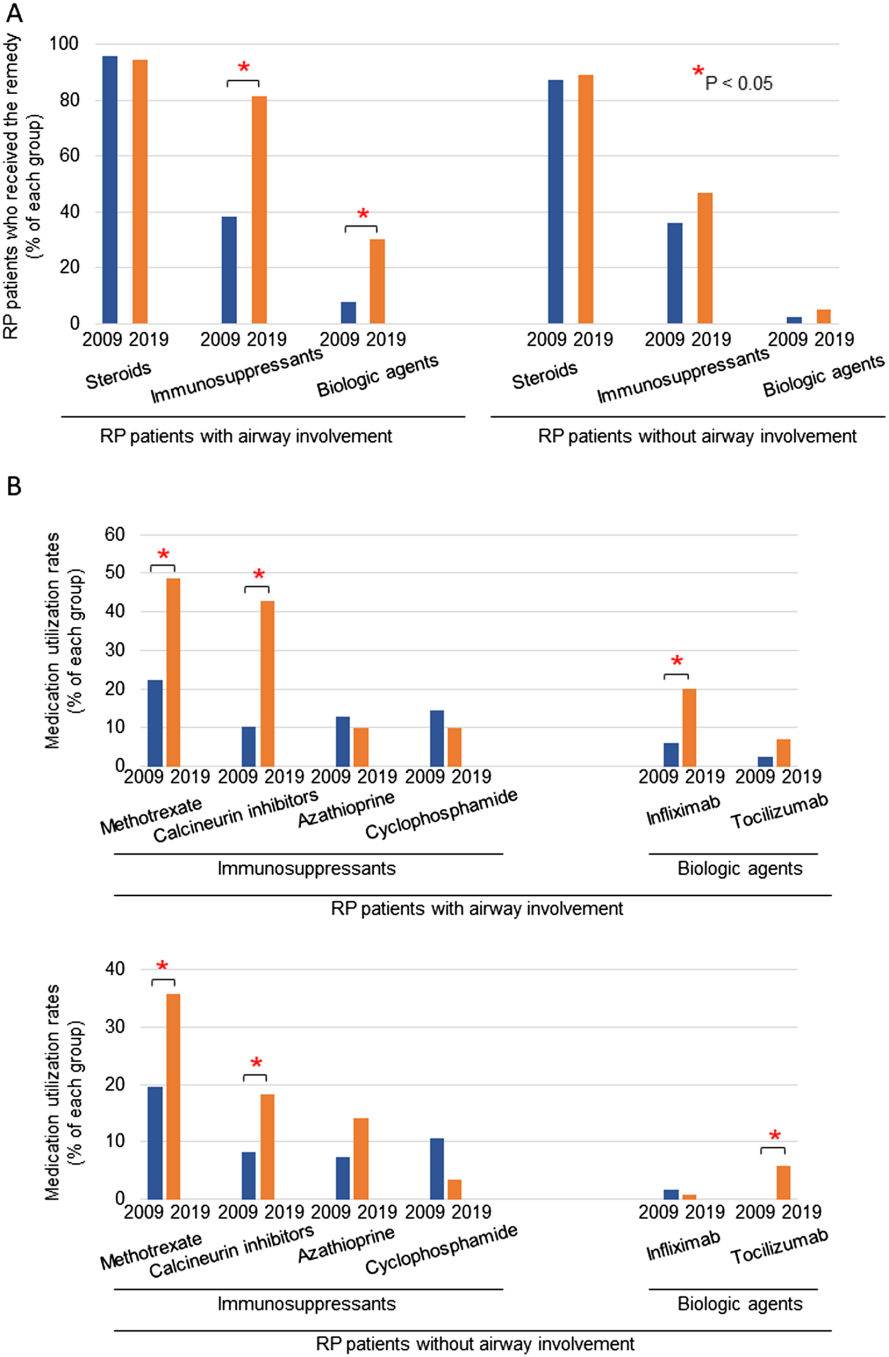
Frequent use of immunosuppressants and biologic agents for RP patients with airway involvement in 2019. Patients were consisted of two groups, patients with airway involvement and those without airway involvement. (**A**) We evaluated utilization rates of steroids, immunosuppressants, and biologic agents in 2009 survey and 2019 survey. Utilization rates of steroids were comparable between 2009 and 2019 surveys in both patients with airway involvement and in those without. Biological agents and immunosuppressants were frequently used in 2019 survey compared with those in 2009 survey (p < 0.05). Significantly increased use of immunosuppressants and biologic agents in 2019 survey was observed in RP patients with airway involvement. In RP patients without airway involvement, use of steroids, immunosuppressants, and biologic agents in 2019 survey was comparable to that in 2009 survey. (**B**) In RP patients with airway involvement, we found significantly increased use of methotrexate, calcineurin inhibitors, and infliximab in 2019 survey compared with those in 2009 survey. In RP patients without airway involvement, we found significantly increased use of methotrexate, calcineurin inhibitors, and tocilizumab in 2019 survey compared with those in 2009 survey.

Steroid use of 2019 survey was comparable to that of 2009 survey in both patients with airway involvement and those without (Fig. 1A).

Biological agents and immunosuppressants were frequently used in patients with and without airway involvement in 2019 survey compared with those in 2009 survey (Fig. 1A). We wanted to find characteristics of each immunosuppressant (methotrexate, calcineurin inhibitors, azathioprine, and cyclophosphamide) and biologic agent (infliximab and tocilizumab) when applied onto RP patients with airway involvement and those without (Fig. 1B).

In patients with airway involvement, we found increased use of methotrexate, calcineurin inhibitors, and infliximab in 2019 survey (Fig. 1B). 30 patients with airway involvement (43% of 70 patients; 23 patients with cyclosporine and the remaining 7 patients with tacrolimus) were treated with calcineurin inhibitors in 2019 survey. With regard to 15 patients treated with infliximab, 14 patients had airway involvement (Table 2 and Fig. 1B), suggesting a vast majority of those with severe airway involvement was treated with infliximab.

**Table 2:** Clinical characteristics of RP patients treated with infliximab in 2019 survey (n = 190).

	Patients treated with infliximab (n = 15) (%)	Patients treated without infliximab (n = 175) (%)	P-value
Ear	60.0	85.1	0.013*
Nasal	40.0	25.1	0.21
Inner ear	33.3	21.7	0.3
Joint	33.3	48.0	0.28
Eye	20.0	45.1	0.06
Airway	93.3	32.0	< 0.001*
Skin	0.0	6.3	0.32
Cardiovascular	6.7	8.6	0.83
CNS	0.0	3.4	0.47
Renal	13.3	7.4	0.42

*Significant differences were observed (P < 0.05).

In RP patients without airway involvement, we found increased use of methotrexate, calcineurin inhibitors, and tocilizumab in 2019 survey compared with those in 2009 survey (Fig. 1B). 22 patients without airway involvement (18% of 120 patients; 9 patients with cyclosporine and the remaining 13 patients with tacrolimus) were treated with calcineurin inhibitors in 2019 survey.

Use of azathioprine and cyclophosphamide was comparable between 2019 and 2009 survey regardless of airway involvement (Fig. 1).

## Discussion

We conducted the second nationwide epidemiological survey of patients with RP in 2019. Because airway complications were major causes of mortality in patients with RP^2–4,9^, we first focused on the lesions. The number of RP patients with airway involvement who had immunosuppressants and/or biologic agents increased in 2019 survey (60/70 patients with airway involvement, 85.7%) compared with those in 2009 survey (46/117 patients with airway involvement, 39.3%, P < 0.001).

Similarly, 5 patients (2.6% of recruited 190 patients and 7.1% of patients with airway involvement) in 2019 survey underwent tracheostomy and/or stenting, whereas 48 patients (20% of recruited 239 patients and 41% of patients with airway involvement, P < 0.001) did in 2009 survey (Table 1). We reported that patients treated with prednisolone alone developed airway involvement more frequently compared with patients treated with both steroids and immunosuppressants^5^. At that time, use of biological agents was not widespread^5^. We think that prevention of respiratory lesions became more effective possibly with appropriate treatment including immunosuppressants and/or biological agents.

Recent findings demonstrated that use of biologic agents induced therapeutic responses in RP respiratory complications with the response rates of approximately 60–70%^7,10,11^. TNF inhibitors were reported to induce complete response frequently (defined as no clinical activity) in RP patients during the first 6 months of the administration compared with the other biologic agents^10^, suggesting less severe destruction of airway by TNF inhibitors. In this study, increased use of infliximab was observed in RP patients with airway involvement, but not in those without. Conversely, frequent use of infliximab was not observed with regard to other organs in 2019 survey (Table 2). It is possible that immunosuppressants and biologic agents, including infliximab, have not only achieved significant clinical responses as have been reported by others but also contributed to reduced mortality rate in RP patients with airway involvement shown here. Further studies are needed to elucidate the underlying molecular mechanism of RP airway involvement for more effective prevention of the involvement.

Cardiovascular involvement^2–4,6,12^ and neurological involvement^2,4,13^ were severe clinical conditions in the patients. We found that incidence of neurological involvement decreased in 2019 survey (Table 1), suggesting beneficial effects of immunosuppressants and/or biologic agents on neurological involvement, as airway involvement.

In contrast, incidence of cardiovascular involvement in 2019 survey was comparable between the two surveys (Table 1). Postoperative use of steroids was reported to associate with postsurgical valvular complications in patients with RP^12^. Indeed, cardiovascular involvement of RP was suggested to be difficult in its management^12^. A larger cohort study, such as a worldwide study, may enable us to accurately determine a most appropriate use of medication in RP patients with cardiovascular involvement. In parallel, it is needed to elucidate the underlying molecular mechanisms of cardiovascular involvement for more effective prevention and treatment.

Calcineurin inhibitors are standard second-line therapies for RP^14,15^. In 2019 survey, 30 RP patients with airway involvement received calcineurin inhibitors. Calcineurin inhibitors may be effective for the treatment and prevention of RP airway involvement as well.

As with the majority of retrospective studies, the design of the current study is subject to limitations. We performed our 2009 and 2019 surveys using a physician questionnaire to control for systemic biases but this type of studies were potentially affected by selection biases. As above mentioned, we estimated that the patient number was 400–500 in 2009^5^. Previous population-based studies demonstrated the similarity in the prevalence and incidence of RP across countries^14,16,17^. Based on the data, we suggest that the number of RP patients is not changed significantly in Japan during the decade. Further prospective studies are needed to confirm our findings of this study, especially the data of disease incidence and mortality.

Collectively, during this decade, incidence of airway involvement and mortality decreased significantly in patients with RP in Japan. Increased use of infliximab in 2019 survey was observed in RP patients with airway involvement, but not in those without. Infliximab administration may become a choice for the treatment of RP patients with airway involvement.

## Methods

### A multi-institutional epidemiological study of RP patients in 2019 (2019 survey)

We utilized a physician questionnaire, the same form as 2009 survey^5^, for 2019 survey. In our preliminary survey, we sent the questionnaire to 5118 major medical facilities and outpatient clinics to assess whether the physicians have been treating or treated at least one patient with RP. Then we sent the second questionnaire to 90 major medical facilities and 287 outpatient clinics. We anonymously collected patient data from 52 major medical facilities (response rate, 58%) and 15 outpatient clinics (5.2%). We suggest that the difference of survey response rates may depend on their data management system. We did not find any statistical differences in the demographic data, clinical features, and medications between hospital outpatients and clinic outpatients. Based on the data, we suggested that 20–30% patients were enrolled in both surveys.

The questionnaire consisted of 5 sections as follows: (1) patients' profiles, (2) clinical features, (3) laboratory findings, (4) treatment, and (5) prognosis. In the clinical feature section, physicians examined the involved organs by assigning into 10 items, that is, (1) ear, (2) nasal, (3) inner ear, (4) joint, (5) ocular, (6) airway, (7) skin, (8) cardiovascular, (9) neurological, and (10) renal involvement.

In the medical treatment section, there were 3 major categories, that is, (1) steroids, (2) immunosuppressants, and (3) biologic agents.

The prognosis section consisted of 5 items, that is, (1) no medication, (2) well-controlled, (3) limited responses, (4) progressive disease course, and (5) death.

This study was approved by the institutional review boards of St. Marianna University School of Medicine (approval number 2406) and was registered with the University Hospital Medical Information Network-Clinical Trials Registry (UMIN000018937). We used an anonymous physician questionnaire for 2009 survey^5^ and 2019 survey (current study). The Ministry of Health, Labor and Welfare of Japan waived informed consent for the anonymous physician questionnaire-based studies in both 2009 and 2019^18^. We conducted our research according to the principles expressed in the Declaration of Helsinki. This cross-sectional study conforms to the STROBE (The Strengthening the Reporting of Observational Studies in Epidemiology) guideline and the CROSS (The Consensus-Based Checklist for Reporting of Survey Studies) guideline.

### Statistical analysis

We compared age, age at onset, and disease duration, using Wilcoxon rank sum test. We compared gender and mortality rates using Fisher's exact test. We utilized dummy variables for the comparison of the incidence of organ involvement and medication use with Wilcoxon rank sum test. The values 0 and 1 represented the absence and presence, respectively, of organ involvement and medication use. Several parameter titers were expressed as mean plus/minus standard error of the mean. P-value less than 0.05 was considered significant. We used software JMP 13.0.0 (SAS Institute Japan, Tokyo, Japan) for statistical analysis.

## Data Availability

The data underlying this article will be shared on reasonable request to the corresponding author.
